# Effectiveness of delivering integrated COPD care at public healthcare facilities: a cluster randomised trial in Pakistan

**DOI:** 10.3399/bjgpopen18X101634

**Published:** 2019-03-20

**Authors:** Muhammad Amir Khan, Nida Khan, John D Walley, Muhammad Ahmar Khan, Joseph Hicks, Maqsood Ahmed, Faisal Imtiaz Sheikh, Muhammad Ali, Farooq Manzoor, Haroon Jehangir Khan

**Affiliations:** 1 Chief Coordinating Professional, Association for Social Development, Islamabad, Pakistan; 2 Project Coordinator, Association for Social Development, Islamabad, Pakistan; 3 Professor of International Public Health, Nuffield Centre for International Health and Development, University of Leeds, Leeds, UK; 4 Research Coordinator, Association for Social Development, Islamabad, Pakistan; 5 Senior Medical Statistician, Nuffield Centre for International Health and Development, Leeds Institute of Health Sciences, University of Leeds, Leeds, UK; 6 Senior Professional, Association for Social Development, Islamabad, Pakistan; 7 Research Coordinator, Association for Social Development, Islamabad, Pakistan; 8 Research Assistant, Association for Social Development, Islamabad, Pakistan; 9 Provincial Manager, Non-Communicable Disease Control Program, Directorate General of Health Services, Punjab, Pakistan; 10 Director, NCD & Mental Health, Directorate General of Health Services, Punjab, Pakistan

**Keywords:** Pakistan, public health facilities, COPD, integrated care package, primary care, general practice

## Abstract

**Background:**

In Pakistan chronic obstructive pulmonary disease (COPD) prevalence is 2.1% in adults aged >40 years. Despite being a health policy focus, integrated COPD care has remained neglected, with wide variation in practice.

**Aim:**

To assess whether enhanced care at public health facilities resulted in better control of COPD, treatment adherence, and smoking cessation.

**Design & setting:**

A two-arm cluster randomised controlled trial was undertaken in 30 public health facilities (23 primary and 7 secondary), across three districts of Punjab, between October 2014–December 2016. Both arms had enhanced diagnosis and patient recording processes. Intervention facilities also had clinical care guides; drugs for COPD; patient education flipcharts; associated staff training; and mobile phone follow-up.

**Method:**

Facilities were randomised in a 1:1 ratio (sealed envelope independent lottery method), and 159 intervention and 154 control patients were recruited. The eligibility criteria were as follows: diagnosed with COPD, aged ≥18 years, and living in the catchment area. The primary outcome was change in BODE (Body mass index, airway Obstruction, Dyspnoea, Exercise capacity) index score from baseline to final follow-up visit. Staff and patients were not blinded.

**Results:**

Six-month primary outcomes were available for 147/159 (92.5%) intervention and 141/154 (91.6%) control participants (all clusters). The primary outcome results cluster-level analysis were as follows: mean intervention outcome = -1.67 (95% confidence intervals [CI] = -2.18 to -1.16); mean control outcome = -0.66 (95% CI = -1.09 to -0.22); and covariate-adjusted mean intervention–control difference = -0.96 (95% CI = -1.49 to -0.44; *P* = 0.001).

**Conclusion:**

The findings of this trial and a separate process evaluation study support the scaling of this integrated COPD care package at primary and secondary level public health facilities in Pakistan and similar settings.

## How this fits in

Integrated COPD care at public facilities is a policy focus and a priority for the Punjab Non-communicable Disease Control Programme. The two key components of the intervention were medication and patient education, including smoking cessation. Effectiveness of delivering integrated COPD care at public health facilities has never been evaluated before in Pakistan. Evidence was required for the programme to take informed intervention scaling decisions.

## Introduction

COPD was found to be the fifth leading cause of death worldwide in 2002, and, if a similar trend continues, it is expected to become the third leading cause of mortality in 2030.^1^ According to World Health Organization, 65 million people suffer from moderate-to-severe COPD, and 90% of the deaths owing to COPD occur in lower- and middle-income countries.^2^ According to an epidemiological survey carried out in 11 countries in the Middle East and North African region, the prevalence of COPD in Pakistan is 2.1% in adults aged >40 years.^3^ This prevalence is expected to increase further given the rising trend in smoking, which is the major risk factor for COPD.^4^

In Pakistan, public funded health care is delivered at primary level through a network of basic health units and rural health centres, and at secondary level through sub-district and district-level hospitals. More than 1000 public healthcare facilities provide free treatment to about 250 000 patients with tuberculosis each year,^1^ and this indicates the feasibility of delivering integrated care for priority health conditions.

In 2003, Pakistan developed a National Action Plan for Prevention and Control of Non-Communicable Diseases, which highlighted the importance of strengthening the delivery of integrated non-communicable disease (NCD) care in the country.^5^ Despite an emphasis in the plan on the integrated service delivery for NCDs, including chronic lung conditions, COPD has remained relatively neglected, and wide variation in diagnosis and treatment practices continues.

Punjab is the country’s biggest province, with a population of 110 million in 36 districts; two-thirds live in rural areas. The available literature^6^ and situation review of primary and secondary level public facilities, which was conducted to inform the intervention design, indicated the following: missing care protocols and staff training; unavailability of essential material inputs (for example, inhalers and peak flow meters); and wide variations in diagnosis and treatment practices.

Therefore, a contextualised intervention package was developed for delivering integrated COPD care at primary and secondary level public healthcare facilities. The intervention package included contextualised care protocols; doctor and allied staff training (carried out jointly by trainers from the provincial programme and the Association for Social Development); patient education materials; and availability of inhalers. The effectiveness of the intervention package at improving the control of COPD was evaluated using a cluster randomised controlled trial.

## Method

The trial is reported here according to the CONSORT guideline recommendations. The trial protocol has been previously published.^1^ In the protocol, both COPD and asthma were covered (two diseases with similar symptoms, such as wheeze). However, after diagnosis, those with asthma were managed and followed-up as a separate cohort; therefore, asthma outcome will be reported in a separate trial outcomes article (in preparation). This cluster randomised controlled trial was conducted between October 2014–December 2016. The trial was carried out in Mandi-Bahauddin, Kasur, and Sargodha districts (in the Punjab province). These districts have a combined population of approximately 8.75 million.

In consultation with the three district health offices, all 32 rural health centres and 9 sub-district hospitals were listed, assessed, and found eligible to participate on the basis of being functional (that is, having basic clinical and laboratory services in place). From the list of 41 eligible facilities, the required number of facilities (*n* = 30) were selected randomly without stratification (resulting in 23 rural health centres and seven sub-district hospitals being selected), and consent for their participation was obtained from the respective district health offices. Then, with the facilitation of staff at each selected facility, the communal consent of the catchment population was obtained from the local community leaders, which included representatives from the women's council, religious leaders, businessmen, teachers, press reporters, and health representatives of the union council.

A cluster design was chosen for this study. This is because it would have not been viable to expect the provider to effectively give or withhold the components of intervention based on patients' allocation to treatment, and there could be possible risk of contamination among the patients. The study only included outpatient departments, which have general doctors who see unreferred primary care patients. Patients were eligible if they were newly diagnosed with COPD and this was based on the guidelines of the Global Initiative for Chronic Obstructive Lung Diseases, which are as follows:^7^ aged ≥18 years old, and currently residing (and expected to continue residing for the next 12 months) in the catchment area of the respective health facility. All the patients attending trial facilities who met the criteria and consented to participate were recruited into the trial between 18 July 2015–10 March 2016. Excluded patients were those who fulfilled the inclusion criteria but refused to participate in the trial. Informed consent was obtained from patients using a standard consent form administered by a 'paramedic' member of health staff (a qualified technical staff member with training in general medical care provision) at the respective healthcare facility.

### Randomisation and blinding

The selection and randomisation of facilities into intervention and control arms (in a 1:1 ratio) was done at the central trial unit of the Association for Social Development, and was monitored by the trial steering committee. The selection of the 30 trial facilities was carried out by listing all 41 eligible facilities in sealed opaque envelopes before shuffling and randomly selecting 30 of them. Then randomisation of the selected facilities (after obtaining district and communal consent) was done by again placing their names into sealed opaque envelopes and shuffling them, before a staff member of the provincial directorate randomly picked 15 envelopes for each treatment arm and opened them. Owing to the nature of the trial, it was not possible to blind individual patients or healthcare providers, but the data analyst was blinded to the treatment allocation.

### Procedures

The intervention arm facilities were provided with contextualised care protocols and tools, 2-day training of doctors and allied staff on full set of care tasks, and materials including inhalers and mobile phones. However, to enable diagnosis and a minimum level of record-keeping, minimal inputs were provided (for example, 1-day staff training on limited care tasks, and material for diagnosis and recording) over and above usual care in control arm facilities. Box 1 gives a summary of inputs in the two arms.

Box 1. **Summary of inputs at intervention and control arm facilities**

**Table:** 

A	B	C
Inputs	Intervention arm facilities	Control arm facilities
Contextualised care protocols and tools	Case management desk guide and counselling tool	None
Training of doctors and allied staff (jointly by the programme staff and research team)	Full care tasks: screen on the first visit, diagnose, and maintain patient records; use provided desk guide on how to prescribe, educate, follow-up, and retrieve patients	Limited care tasks: screen on the first visit, diagnose, and maintain patient records only
Material inputs	Peak flow meter, recording tools; also salbutamol and ipratropium inhalers, mobile reminders for patient retrieval	Peak flow meter, and recording tools only

Access to spirometry at baseline (that is within 2 weeks of registration) and endline (that is completed 6 months after registration) was offered as a research measurement activity (that is not to inform clinical decisions) for all patients in the two trial arms.

In both arms, the doctors and allied staff were enabled to screen (on the first visit) and diagnose patients with COPD. They could also maintain the chronic disease card for each patient (with a line to record clinical changes per attendance). Additionally, in the intervention arm, the facility staff were enabled to enhance COPD treatment and follow-up care; for example, they were able to prescribe drugs according to guidelines; educate patients with the help of the pictorial flipchart on preventive measures, including smoking cessation; dispense free-of-charge inhalers (salbutamol and/or ipratropium bromide); add or amend drugs as required during the monthly follow-up; and identify and retrieve patients with delayed monthly follow-up visits, using mobile phone messages or calls.

Further details of lung healthcare experiences are given in the process evaluation study already published in this journal,^8^ and the guides and tools are available at http://comdis-hsd.leeds.ac.uk/resources/ncd-care-package.

### Data collection and outcomes

The patient data at the baseline and subsequent five monthly follow-up visits were recorded on the chronic disease card as part of the care delivery. The facility doctor recorded the clinical data (that is, the diagnosis and prescription); 'paramedic' staff recorded basic data (for example, name, age, sex, weight, height, peak expiratory flow rate result, and residential address). Patients who did not turn up for their follow-up visit were declared lost to follow-up if they could not be traced and did not return for follow-up within 2 months of their last visit due date. The BODE index score,^9,10^ which is a composite measure for assessing the severity of disease symptoms, was used to measure the outcomes in all patients with COPD. A score of 0–2 was chosen as ‘good control’ because this represents the best 80% 4-year survival, whereas a score of 3–4 is 67%, and 5–6 is 57% (a 1-point change is a 5%–6% reduction in 4-year survival).^11^ The spirometry was performed, as an additional research activity, by an external assessor, who was a qualified public health professional with hands-on training by a chest specialist at a teaching hospital. The test results were retrieved directly from the data stored in the spirometer. The primary outcome was the mean change in patient BODE index score from baseline to 6-month follow-up (calculated as 6-month value minus baseline value). The patients received points ranging from 0 (lowest value) to 3 (maximal value) for each threshold value of FEV_1_, distance walked in 6 minutes, and score on the modified Medical Research Council (mMRC) dyspnoea scale.^12^ For body mass index, the values were 0 or 1. The points for each variable were added, so that the BODE index score for each patient ranged from 0–10 points. Box 2 gives BODE index scoring details.

Box 2. **BODE index scoring**

**Table:** 

**Clinical feature**	**BODE index score points**			
	**0**	**1**	**2**	**3**
mMRC scale	0–1	2	3	4
6MWD, m	≥350	250–349	150–249	≤149
FEV_1_% pred	≥65	50–64	36–49	≤35
BMI, kg/m^2^	>21	≤21		
**mMRC dyspnoea scale ranges from 0–4:** Score 0–1 indicates breathlessness on exercise only, or on brisk walking Score 2 indicates person walks slowly or stops for breath (due to breathlessness) Score 3 indicates person stops for breath in less than 100 m walking Score 4 indicates person is breathless even while dressing **Distance walked in 6 minutes, contributes 0–3 in the BODE index score. Patient is asked to walk for 6 minutes and his/her score is calculated as below:** Score 0 indicates ≥350 m walking Score 1 indicates 250–349 m walking Score 2 indicates 150–249 m walking Score 3 indicates ≤149 m walking **The spirometry (FEV_1_% pred.) contributes 0–3 in the BODE index score:** Score 0 ≥65 FEV_1_% pred. (FEV_1_ is 65% or more of the predicted amount) Score 1 ≥50-64 FEV_1_% pred. (FEV_1_ is 50–64% of the predicted amount) Score 2 ≥36-49 FEV_1_% pred. (FEV_1_ is 36–49% of the predicted amount) Score 3 ≤35 FEV_1_% pred. (FEV_1_ is 35% or less of the predicted amount) **Body mass index (relates height with weight), contributes 0–1 in the BODE index score** Score 0 indicates BMI >21 Score 1 indicates BMI ≤21				

Extracted from the doctors’ training module, developed as part of intervention.

6MWD = six-minute walk distance. BODE = Body mass index, airway Obstruction, Dyspnoea, Exercise capacity. BMI = body mass index. mMRC = modified Medical Research Council.

The three secondary outcomes, which were all added post-protocol, were as follows: (a) COPD control was classed as controlled if a patient’s BODE index score was 0–2, and uncontrolled if it was 3–10 at 6-month follow-up; (b) smoking status as given by patient when asked by an external assessor (smoking or not smoking) at 6 months; and (c) follow-up adherence, which was defined as at ≥3 of the five required follow-up facility visits made and/or recorded within the 6-month intervention period.

All data were entered into the SPSS (version 20) database. The quality of data entry was assured through training of data-entry personnel and checking the quality of data entered at regular intervals.^13^

### Sample size calculation

At the time of design, the sample size requirement was estimated at a total of 18 clusters and 306 patients with COPD. The sample size was required to detect a mean change of 1 point in the BODE index score (that is, from the assumed 4 at the baseline), with 80% power, based on a two-tailed hypothesis test with a 5% significance level. This assumed a standard deviation of 2.0; cluster size of 17; and intracluster correlation coefficient (ICC) of 0.05.

In the initial piloting of the intervention, it was learnt that the assumed rate of patient registration in each cluster was higher than the actual registration. In light of the piloting, the trial design was revised and the total number of clusters was increased from 18 to 30 so that the required total number of patients could be registered, followed, measured, and analysed within the time constraints.

### Statistical analysis

To analyse the data, robust methods (suitable for cluster trials with relatively few clusters per arm) were used.^14^ For the continuous primary outcome, a crude analysis was initially carried out by calculating cluster-level outcome values based on the mean of all outcome scores in each cluster. An independent *t*-test was then used to estimate the treatment effect as the mean difference in the cluster-level outcome values between treatment arms (intervention minus control), with the associated 95% CI and *P* value. To adjust for potentially confounding covariates, a two-stage approach was used. First, a linear regression model was fitted to the individual-level outcome data to adjust for covariates of interest, but excluding the treatment effect. A covariate adjusted difference-residual for each cluster was then calculated from the model, by calculating the mean difference between the observed and model predicted outcomes for each cluster. An independent *t*-test was then used to estimate the covariate-adjusted treatment effect as the mean difference in the cluster-level difference-residuals between treatment arms, with the associated 95% CI and *P* value.

The three secondary binary outcomes were analysed similarly. A crude analysis was first done by calculating cluster level proportions, and an independent *t*-test was then used to estimate the treatment effect as the risk difference (that is, absolute difference in outcome proportions) between the intervention and control arms, with the associated 95% CI and *P* value. To adjust for covariates, the same two-stage approach was used, but with a logistic regression model used instead to calculate the cluster-level difference residuals, which were then analysed using an independent *t*-test as described above.^14^

All patients and clusters were analysed according to their original treatment allocations, and complete-case analyses were conducted, excluding any patients from analyses where their outcome and/or covariate data were missing, depending on the data required in the analysis. Statistical significance was set at the 5% level and two-sided *P* values were calculated. There were no interim analyses planned or conducted. The ICC was estimated from the variance component of a one-way analysis of variance mode using the R-package ICC.^15^

## Results

**Figure 1.**
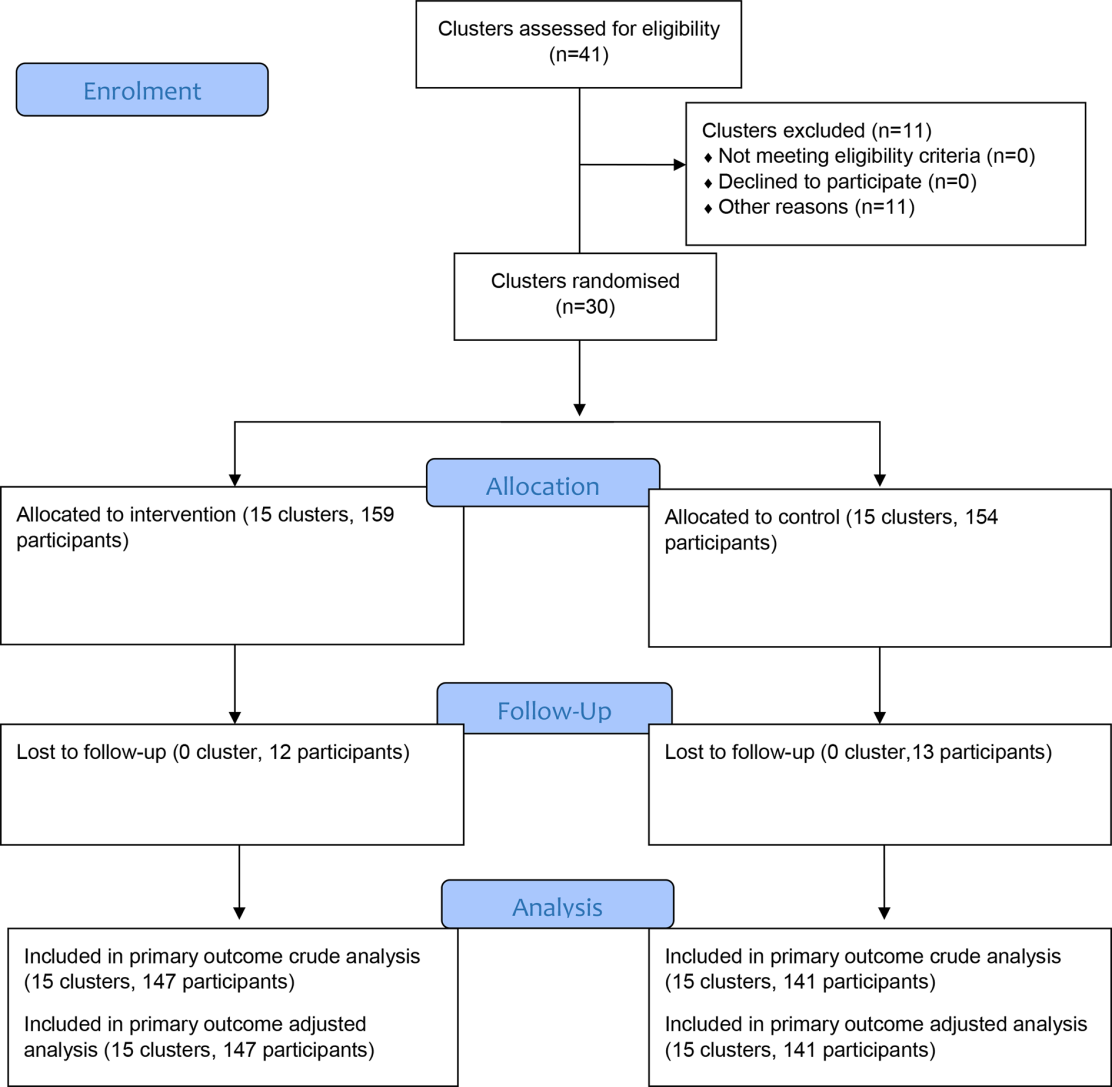
CONSORT trial flow chart

A total of 15 health facilities were randomised to each arm in October 2014, and 313 patients were recruited between 18 July 2015–10 March 2016. All follow-ups were completed by 30 September 2016. The trial flow is shown in Figure 1. The baseline characteristics of individuals in both arms appeared to be well balanced (Table 1). The patient loss to follow-up rate was modest and similar in both the arms, with 7.5% in the intervention arm and 8.4% in the control arm.

**Table 1.** Baseline characteristics

**Characteristics**	**Intervention, *n* (%)**	**Control, *n* (%)**
**Clusters**		
Total	15	15
**Doctors**		
Male	15 (100.0)	15 (100.0)
Female	0 (0.0)	0 (0.0)
**Paramedics**		
Male	15 (100.0)	15 (100.0)
Female	0 (0.0)	0 (0.0)
**Participants**		
Total	159 (50.8)	154 (49.2)
Mean cluster size, ±SD	10.60 ± 3.85	10.27 ± 3.90
Male	122 (76.7)	111 (72.1)
Female	37 (23.3)	43 (27.9)
Mean age, years, ±SD	48.11 ± 13.89	48.47 ± 12.86
Not educated	104 (70.7)	102 (72.3)
Primary (grade 1–5)	16 (10.9)	15 (10.6)
Secondary (grade 6–12)	24 (16.3)	22 (15.6)
Above secondary (grade >12)	3 (2.0)	2 (1.4)
Mean BMI, kg/m^2^, ±SD	22.47 ± 4.79	22.45 ± 5.93
Diagnosed with COPD	159 (100.0)	154 (100.0)
Smoker	59 (37.11)	54 (35.06)
Mean BODE index score, ±SD^a^	3.85 ± 1.94	3.78 ± 1.88
Mean PEFR value, ±SD	232.97 ± 100.88	246.81 ± 108.73
Mean weight, kg, ±SD	58.69 ± 13.18	58.24 ± 14.09
Mean height, inches, ±SD	63.68 ± 3.83	63.63 ± 3.85

^a^Spirometry was done by an external assessor within 15 days of diagnosis and/or registration.

BODE = Body mass index, airway Obstruction, Dyspnoea, Exercise capacity. BMI = body mass index. COPD = chronic obstructive pulmonary disease. PEFR = peak expiratory flow rate. SD = standard deviation.

Between baseline and five monthly follow-up visits, the BODE Index improved (that is, the score reduced) in both treatment arms, but there was a statistically and clinically significantly greater reduction in the intervention arm compared with the control arm (adjusted difference -1, 95% CI = -1.5 to -0.4; *P* = 0.001). There was also a statistically and clinically significantly greater percentage of COPD control at 6-month follow-up in the intervention arm compared with the control arm (adjusted difference 29 percentage points, 95% CI = 12.4 to 45.6; *P* = 0.001). There was also a statistically and clinically significantly higher quit rate among smokers at 6-month follow-up in the intervention arm compared with the control arm (adjusted difference 32 percentage points, 95% CI = 15.4 to 48.5; *P* =0.001). Lastly, there was a statistically and clinically significantly greater level of follow-up adherence in the intervention arm compared with the control arm (adjusted difference 40.4 percentage points, 95% CI = 24.2 to 56.7; *P*<0.001). Adjusted and crude results were very similar, apart from for the quit rate among smokers outcome, where the crude result showed a slightly greater quit rate among smokers (see Table 2).

For all crude analyses, except for the change in smoking, data were available for 147/159 (92.5%) participants in the intervention arm and for 141/154 (91.6%) participants in the control arm. For the quit rate among smokers, crude analysis data were available for 56/59 (94.9%) participants in the intervention arm and 50/54 (92.6%) participants in the control arm. For all the adjusted analyses, except for quit rate among smokers, data were available for 147/159 (92.5%) participants in the intervention arm and 141/154 (91.6%) participants in the control arm. For the adjusted analysis of the change in smoking outcome data were available for 56/59 (94.9%) in the intervention arm and 50/54 (92.6%) in the control arm. Unadjusted primary outcome ICC for: (a) overall = 0.25, (b) the intervention arm = 0.05, and (c) the control arm = 0.21.

**Table 2.** Primary and secondary outcomes

	Mean outcome (95% CI)^a^		Crude intervention- control difference (95% CI); *P* value^b^	Adjusted intervention- control difference (95% CI); *P* value^b^	
	Intervention (clusters = 15)	Control (clusters = 15)		
**Primary outcomes**				
BODE index score change^c^	-1.67 (-2.18 to -1.16)	-0.66 (-1.09 to -0.22)	-1.01 (-1.65 to -0.37); 0.003	-0.96 (-1.49 to -0.44); 0.001
COPD control^d^	66.88% (54.99 to 78.77)	38.20% (22.35 to 54.06)	28.68% (9.68 to 47.67); 0.005	29.03% (12.41 to 45.64); 0.001
**Secondary outcome**				
Quit rate among smokers^e^	53.90% (34.98 to 72.82)	17.52% (7.36 to 27.69)	36.38% (15.66 to 57.10); 0.002	31.98% (15.42 to 48.54); 0.001
Follow-up adherence^f^	65.54% (52.64 to 78.44)	25.17% (14.86 to 35.47)	40.38% (24.57 to 56.18); <0.001	40.40% (24.15 to 56.67); <0.001

**^a^**Arm-specific mean outcomes and their 95% confidence intervals are calculated from cluster-level mean and/or proportion summary values of outcomes. ^b^All intervention minus control differences (that is, intervention effect estimates) are based on crude and/or covariate-adjusted analysis of cluster-level mean and/or proportion summary values of outcomes. ^c^BODE index score change calculated as outcome at 6-month follow-up minus outcome at baseline. ^d^COPD control defined as BODE index ≤ 2 at 6-month follow-up. ^e^Quit rate among smokers calculated as smokers who had quit smoking at 6-month follow-up. ^f^Follow-up adherence defined as attending ≥4 follow-up visits. All analyses use only complete cases.

BODE = Body mass index, airway Obstruction, Dyspnoea, Exercise capacity. CI = confidence intervals. COPD = chronic obstructive pulmonary disease.

## Discussion

### Summary

The integrated COPD care package, which included standardised diagnosis, prescription, patient education, free drugs, and follow-up adherence support, offered in the intervention clusters of public health clinics was found to be more effective in terms of achieving the COPD control at 6 months of completed treatment. In the trial, the better control of COPD in the intervention arm was likely to be due to a combined effect of the care components; it was not possible to separate the individual effects of standardised prescription, free-of-cost drug provision, patient education, and follow-up adherence support.

### Strengths and limitations

The main strength of this study was that it was designed and developed to be potentially replicable and sustainable in the routine public healthcare system of Pakistan or in similar settings; and the care and research protocols and tools were piloted before the trial.

The lottery method is known to be a suitable method for randomisation in small populations compared with large populations; therefore, the randomisation was considered adequate for the study.^16^ A methodological limitation of the study was that the control arm had to be strengthened for diagnosis and record-keeping (as in the intervention arm), more than is routine within such public health facilities, to ensure standardisation for research purposes. However, this is likely to have led to a reduction in the observed intervention effect compared with what may have been observed if it had been possible to compare facilities where there had been no interventions at all. This is owing to the influence of better diagnosis and record-keeping components introduced to the control arm. Another limitation was that neither the healthcare providers nor the patients were blinded to intervention or control clusters, which increases the likelihood that there was positive bias introduced into the estimated intervention effect. This is owing to providers and patients being aware of treatment allocation status. The trial also only lasted for a short duration (6 months) relative to the long-term nature of COPD, and so a longer study would be necessary to evaluate how sustainable the improvements in patients’ control of COPD would be.

### Comparison with existing literature

As the COPD care intervention included both medication and patient education components, the reported clinical outcomes (for example, reduction in BODE index score, and adherence to follow-up) were the combined effect of the two components; that is, medication and patient education. The researchers were not able to find published results of any intervention trial where similar clinical outcomes were used to assess the effectiveness of an integrated COPD care at primary and secondary level public health facilities.

An intervention trial on COPD care focusing on inhalation technique and adherence to maintenance therapy reported significantly enhanced inhalation score and medication adherence among patients with COPD.^17^ Other published studies have also highlighted the importance of prescribing inhalers at all stages of the disease;^7^ and educating patients to use inhalers correctly^18^ to help cope with the disease.^19,20^ The BODE index is considered a better measure for classifying the severity of COPD and predicting the change in health-related quality of life.^21^ However, a study highlighted the need for specialist skills to carry out spirometry at primary healthcare level.^22^ In this trial, specially trained staff conducting spirometry at the respective health facilities was considered an acceptable balance of technical requirements and feasibility considerations. The selection and measurement of effectiveness (using BODE index score) is in accordance with the current knowledge of clinical care and its outcomes.

Patient non-adherence to the follow-up visits seems to be a major treatment challenge both in the intervention and control arms (although less so in the intervention compared with the control arm). A process evaluation of the intervention^8^ indicated that patients face social challenges to access care at public facilities (for example, facility timing), and that the provision of free drugs and use of mobile phone messages can be helpful for patient adherence to the follow-up visits.

The ASSIST trial^23^ at public health facilities in Pakistan, reported that simplified, tool-assisted counselling achieved around a 40% smoking quit rate among people with productive cough of ≥2 week duration (that is, patients with suspected tuberculosis). The evidence-based counselling tool used in the ASSIST trial was further adapted and included in the care package for chronic obstructive lung conditions. A relatively higher quit rate (around 54%) achieved through the tool-assisted counselling of COPD patients could either be due to difference in the severity of chest-symptoms (between patients with COPD and suspected cases of tuberculosis) or the rigour of the quit measurement; for example, only asking the patient (as in the COPD trial) or asking the patient *and* testing carbon monoxide (as in the ASSIST trial). Both trials in a low-income setting indicate a potentially high dividend (a 40%–54% quit rate) of counselling smokers with ≥1 chest symptoms.

### Implications for research and practice

The integrated COPD care package for public health facilities has been shown to be effective, leading to improved COPD control outcomes within the low-income country setting of Pakistan.

In light of the evidence from the trial, and the process evaluation study^8^ (published separately), the Department of Health Punjab has decided to do the following: (a) scale-up the COPD care in all 36 districts; and (b) include inhalers in the list of essential drugs and the procurement plans for all public facilities in the province.

Further research is suggested to explore feasible ways to enhance adherence to follow-up visits, such as the use of electronic medical records, and to assess the separate effects of the intervention components, in particular the provision of free drugs.

## Trial registration

Current Controlled Trials reference number: ISRCTN17409338.
